# pH-responsive ZIF-8-based nitazoxanide nanodelivery system for synergistic inhibition of retinoblastoma growth

**DOI:** 10.1039/d6ra04041a

**Published:** 2026-07-30

**Authors:** Mu Qin, Sanhua Xu, Zhengri Li, Yi Shao, Jingxiang Zhong

**Affiliations:** a Department of Ophthalmology, The First Affiliated Hospital of Jinan University Guangzhou 510632 China zjx85221206@126.com; b Department of Ophthalmology, The Affiliated Hospital of Xiangnan University Hunan 423000 China; c Ophthalmic Center, The 2nd Affiliated Hospital, Jiangxi Medical College, Nanchang University Nanchang Jiangxi 330006 China; d Department of Ophthalmology, The Affiliated Hospital of Yanbian University Jilin 133000 China; e Department of Ophthalmology, Shanghai General Hospital, Shanghai Jiao Tong University School of Medicine, National Clinical Research Center for Eye Disease Shanghai 200080 China freebee99@163.com

## Abstract

Retinoblastoma (RB) remains a predominant intraocular malignancy in the pediatric population, and its clinical management is significantly impeded by limited blood-retinal barrier (BRB) penetration, the development of multidrug resistance, and severe systemic toxicity. This study aims to develop a novel nanodelivery system based on Zeolitic Imidazolate Framework-8 (ZIF-8) to overcome the limited bioavailability of the antiparasitic drug nitazoxanide (NTZ), and to enhance its anti-retinoblastoma efficacy. We successfully engineered ZIF-8@NTZ nanoparticles characterized by a robust core–shell architecture. Characterization results showed that the particles exhibited high drug loading capacity (approximately 58.628%), suitable particle size (∼231 nm), and significant pH-responsive drug release properties. *In vitro* assays indicated that ZIF-8@NTZ exerted potent inhibition on RB cell proliferation and induced apoptosis. *In vivo* experiments further confirmed that ZIF-8@NTZ could significantly inhibit tumor growth. This study indicates that the ZIF-8@NTZ nanosystem provides a promising strategy for the targeted therapy of retinoblastoma.

## Abstract 2

ZIF-8@NTZ nanoplatform improves nitazoxanide delivery and suppresses retinoblastoma growth.

## Graphical abstract

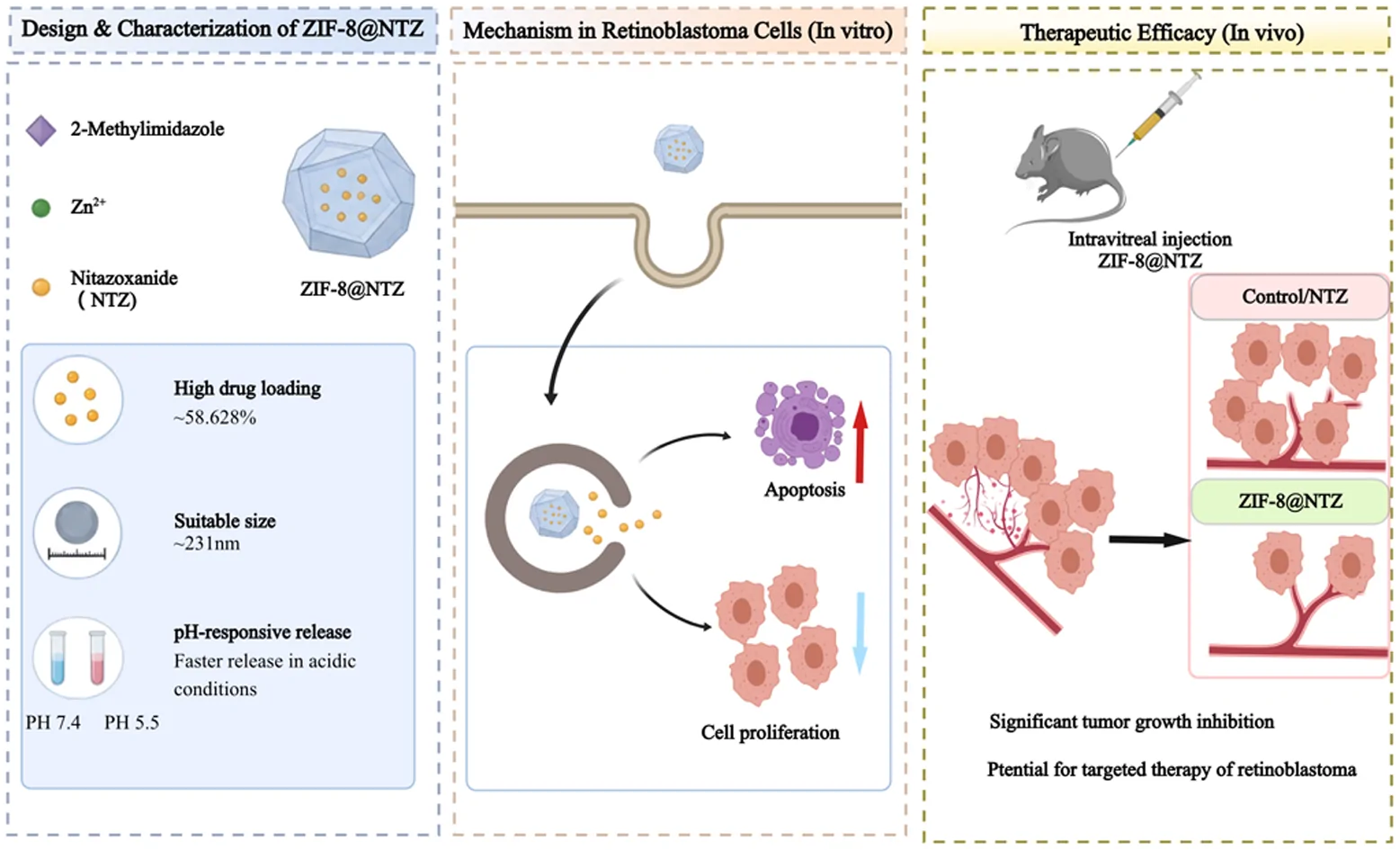

## 1. Introduction

Retinoblastoma (RB) is the most prevalent intraocular malignancy in children aged 0–4 years, accounting for approximately 3–4% of all malignant tumors in this demographic.^1–3^ The global annual incidence is estimated at 1/15 000 to 1/20 000 live births, reaching up to 1/10 000 in regions such as Southeast Asia, Africa, and central and western China.^4^ Due to subtle initial clinical manifestations, such as leukocoria or strabismus, approximately 50% of patients are diagnosed at International Intraocular Retinoblastoma Classification (IIRC) stage D or E, often accompanied by high-risk features such as hyphema, neovascular glaucoma, or optic nerve invasion, leading to an enucleation rate exceeding 60%.^5,6^ Even when eye preservation is successful, 10% of patients may develop optic nerve remnant or choroidal invasion, with distant metastases most commonly found in bone marrow and the central nervous system, with a mortality rate exceeding 50%.^7^

The pathogenesis of RB is primarily driven by biallelic mutations in the RB1 tumor suppressor gene. The encoded Rb protein regulates cell cycle progression, and its dysfunction precipitates malignant transformation.^8^ RB patients require multiple treatments, including focal therapies (cryotherapy^9^ and laser), chemotherapy (intra-arterial chemotherapy), radiotherapy (external beam radiotherapy and plaque brachytherapy), and even enucleation.^10,11^ Among these therapies, intra-arterial chemotherapy has demonstrated significant efficacy for vitreous seeds with minimal retinal toxicity.^12^ However, this approach requires specialized multidisciplinary expertise and advanced medical infrastructure. Moreover, the issues of limited durability and toxicity remain unresolved for many centers.^13,14^ Other serious side effects of these local treatments include cataract, facial deformity, radiation retinopathy, and even the potential risk of secondary tumors.^15^ Currently, intravenous chemotherapy (VEC regimen: vincristine, etoposide, carboplatin) combined with local laser/cryotherapy is the first-line treatment for RB.^16^ Nevertheless, this regimen is often hampered by multidrug resistance (*e.g.*, MDR1/P-gp overexpression) and severe systemic adverse effects, including myelosuppression, ototoxicity, and therapy-related leukemia.^17^ Additionally, the presence of the blood-retinal barrier (BRB) results in drug concentrations in the vitreous and subretinal fluid being only 5–10% of plasma levels, while repeated intravitreal injections increase the risk of retinal detachment, hemorrhage, and endophthalmitis.^18^ Therefore, there is an urgent need to develop a novel drug delivery system that can achieve targeted drug delivery, reduce side effects, and improve therapeutic efficacy.

Nitazoxanide (NTZ) is an FDA-approved antiparasitic agent that is rapidly deacetylated to form its active metabolite tizoxanide (TIZ).^19^ Recent studies have found that NTZ exhibits nanomolar-level antitumor activity in colorectal cancer by modulating the Wnt/β-catenin signaling pathway and activating the ROS-p53 axis.^20^ Our previous experimental results showed that the half-maximal inhibitory concentration (IC50) of NTZ against retinoblastoma Y79 and WERI-Rb-1 cell lines between 15–20 µM, significantly lower than that of carboplatin (IC50 ≈ 50 µM). However, the extremely low water solubility of NTZ (<5 µg mL^−1^ at 37 °C), limited oral bioavailability, and less than 1% BRB penetration rate severely limit its intraocular application.^20^

Zeolitic Imidazolate Framework-8 (ZIF-8), formed by rapid coordination of biocompatible Zn^2+^ ions with 2-methylimidazole in aqueous solution at room temperature, can achieve high drug loading in one step, effectively solving the poor water solubility of NTZ.^21,22^ Its particle size of 80–90 nm and surface zeta potential of approximately +25 mV give it good enhanced permeability and retention (EPR) effects, allowing it to passively accumulate in tumor tissues.^23^ More importantly, ZIF-8 can rapidly disintegrate under weakly acidic conditions (pH 5.0), a characteristic that ensures “burst” release of drugs in the tumor microenvironment while remaining stable in the normal physiological environment (pH 7.4) with low leakage rate.^24^ The liberated Zn^2+^ ions may further synergistically elevate intracellular reactive oxygen species (ROS) levels, thereby amplifying the ROS-p53-mediated antitumor pathway of NTZ.^25,26^ Based on the above background, this study aims to construct a core–shell ZIF-8 nanocarrier (ZIF-8@NTZ) to achieve efficient drug loading and pH-responsive release, ultimately enhancing therapeutic efficacy in RB.

## 2. Materials and methods

### 2.1 Materials

Nitazoxanide (NTZ, purity >98%) was obtained from MCE (USA). Zinc nitrate hexahydrate (Zn(NO_3_)_2_·6H_2_O) and 2-methylimidazole (2-MIM) were purchased from Sigma-Aldrich (USA). Human retinoblastoma cell lines (Y79 and WERI-Rb-1) were sourced from the American Type Culture Collection (ATCC). All cell culture reagents were provided by Gibco company. A live/dead cell viability assay kit was purchased from Beyotime (Jiangsu, China). The Annexin V-FITC/PI apoptosis detection kit was purchased from BD (USA) and the EdU proliferation assay kit was purchased from Beyotime (Shanghai, China).

### 2.2 Characterization

Morphological analysis was performed using transmission electron microscopy (TEM; JEM-2100F, JEOL, Japan) and scanning electron microscopy (SEM; JSM-7500F, JEOL, Japan). The dynamic light scattering (DLS) measurements for hydrodynamic diameter and zeta potential were performed using Malvern Zetasizer Nano ZS90 (Malvern Instruments limited, UK). Absorbance was determined using UV-1780 UV-Vis spectrophotometer (SHIMADZU, Japan). Contact angles were measured by OCA15EC (DataPhysics Instruments, Germany). FITC-labeled images, live/dead fluorescence staining images of bacteria and cells were captured using Leica DMi8 fluorescence microscope (Leica, Germany). Images of biofilm were taken from TCS-SP8 confocal laser scanning microscope (Leica, Germany). Cell apoptosis experiments were performed by Accuri C6 Plus (BD, USA). Fluorescence images of H&E and immunofluorescence staining experiment corneal sections were captured using Leica DM4B fluorescence microscope (Leica, Germany). The drug loading capacity of the nanocarriers was determined by UV-visible spectrophotometry. ZIF-8, ZIF-8@LEVO, and ZIF-8@LEVO@HA solutions were prepared in deionized water at 0.1 mg mL^−1^, respectively. The absorption spectra of the samples were measured using quartz cuvettes in the range of 200–400 nm with a step size of 1 nm. To evaluate the transparency of CL^−^, the original CL, PEI-CL, and ZLHP-CL were placed vertically and attached to the 2/3 position of the quartz cuvette. The absorption spectra of the lenses were measured in the range of 300–800 nm with a step size of 1 nm.

### 2.3 Preparation of ZIF-8 NPs

ZIF-8 nanoparticles were synthesized by dissolving 0.297 g of Zn(NO_3_)_2_·6H_2_O in 10 mL of methanol (Solution A) and 0.657 g of 2-MIM in 10 mL of methanol (Solution B) under sonication for 10 min. The solutions were combined and stirred at 500 rpm for 10 h at room temperature. After completion, centrifuge at 10 000 rpm, 4 °C for 10 min, discard the supernatant, and then wash with methanol 3 times. Then freeze-dry for later use. The dried white powder is ZIF-8 NPs.

### 2.4 Preparation of nitazoxanide

A stock solution of NTZ (0.25 mg mL^−1^) was prepared by dissolving 25 mg of the compound in a 100 mL volumetric flask with methanol (Solution C). This solution was filtered through a 0.45 µm membrane and degassed *via* sonication prior to experimental application.

### 2.5 Synthesis of ZIF-8@NTZ

Simultaneously pour the above Solutions A, B, and C into a mixed solution and stir (500 rpm, 10 h, room temperature). After completion, centrifuge at 10 000 rpm, 4 °C for 10 min, discard the supernatant, and wash with methanol 3 times to obtain the product.

### 2.6 EdU cell proliferation assay

Logarithmic phase Y79 and WERI-Rb-1 cells were seeded in 96-well plates at 2 × 10^4^ per well. The nitazoxanide mother solution was sterilized with a 0.22 µm filter and diluted to 10, 20, 40 µM with complete medium (final methanol concentration ≤0.1%); the control group received an equal volume of solvent. After 48 h of drug treatment, 50 µM EdU working solution was added to each well and incubated for another 2 h. The medium was discarded, and cells were fixed with 4% paraformaldehyde at room temperature for 15 min, permeabilized with 0.3% Triton X-100 for 15 min. According to the Cell-Light EdU Apollo 567 kit (RiboBio), the click reaction was performed, and nuclei were stained with Hoechst 33 342 (5 µg mL^−1^) for 10 min. Fluorescence inverted microscope 40× objective lens randomly selected 3 fields for photography, and ImageJ counted EdU-positive (red) and total nuclei (blue). The proliferation rate = (EdU^+^cells/total nuclei) × 100%, with six replicate wells per group.

### 2.7 Live/dead cell staining assay

Logarithmic phase Y79/WERI-Rb-1 cells were seeded in 24-well plates at 1 × 10^5^ per well. The NTZ mother solution was sterilized with a 0.22 µm filter and diluted to 10, 20, 40 µM with complete medium (final methanol concentration ≤0.1%); the control group received an equal volume of solvent. After 24 h of drug treatment, cells were gently collected by pipetting, centrifuged at 300 g for 5 min, and resuspended in PBS. An equal volume of AO/PI mixed staining solution (acridine orange 1 µg mL^−1^ + propidium iodide 1 µg mL^−1^) was added, and stained in the dark for 2 min. Immediately take 10 µL of suspension and add to a hemocytometer, photographed with a fluorescence inverted microscope 40× (GFP channel for AO green-stained living cells, Texas-Red channel for PI red-stained dead cells).

### 2.8 Cell apoptosis assay

Logarithmic phase Y79 and WERI-Rb-1 cells were seeded in 24-well plates at 2 × 10^4^ per well. The NTZ mother solution was sterilized with a 0.22 µm filter and diluted to 10, 20, 40 µM with complete medium (final methanol concentration ≤0.1%); the control group received an equal volume of solvent. After 24 h of drug treatment, floating and adherent cells were collected, washed twice with cold PBS, and resuspended in 100 µL binding buffer. Add 5 µL Annexin V-FITC and 5 µL PI, incubate at room temperature in the dark for 15 min, then add 400 µL binding buffer. Take 10 µL and drop on a slide, photographed with a fluorescence inverted microscope 40× (FITC channel for Annexin V green staining, Texas-Red channel for PI red staining).

### 2.9 Western blot

Total protein from cells was extracted with RIPA lysis buffer (Beyotime, P0038). Equal denatured proteins were resolved *via* SDS-PAGE and transferred to PVDF membranes. Membranes were blocked in 5% skim milk (Solarbio, LP0033B) for 1 h at room temperature, followed by overnight incubation with primary antibodies at 4 °C. After TBST washing, blots were probed with HRP-linked secondary antibodies for 2 h at room temperature. Bands were visualized using ECL substrate (YEASEN, 36208ES60) on a Thermo automatic chemiluminescence imager.

### 2.10 PDX culture and HDST

PDXE model tissues were cultured in 700 µL of tissue medium containing NTZ at concentrations of 2.5, 5, 10, 20, 40, 60, 80, and 100 µmol L^−1^. After 3 days, the supernatant was replaced with fresh medium containing an equal amount of drug. Cell inhibition capacity was detected by CCK8 after 7 days of drug addition.

### 2.11 *In vivo* tumor study

1 × 10^5^ human WERI-Rb-1 cells were subcutaneously injected into the right dorsal side of C57BL/6 mice (female). After 7 days, tumor-bearing mice were randomly divided into 3 groups (*n* = 5 per group): control group, NTZ group, and ZIF-8@NTZ group. When tumors reached 50–100 mm^3^, mice were randomized into three groups (*n* = 5): control, NTZ, and ZIF-8@NTZ. Treatments were administered *via* tail vein injection every other day until day 18.

### 2.12 Hematoxylin and eosin (H&E) staining

Tumor tissues were harvested, fixed in 4% paraformaldehyde, embedded in paraffin, sectioned (4–5 µm), and stained with H&E following standard protocols.

### 2.13 Statistical analysis

All statistical analyses were performed using GraphPad Prism 8.4.3 software. Unless otherwise specified, all data are expressed as mean ± standard deviation. One-way analysis of variance (ANOVA) was used to assess differences between groups. Unpaired two-tailed Student's *t*-test was used to compare means between two groups. Statistical significance was set at *p < 0.05, **p < 0.01, ***p < 0.001, ****p < 0.0001. All experiments were repeated three times.

## 3. Results

### 3.1 Preparation and characterization of ZIF-8@NTZ nanoparticles

**Fig. 1:**
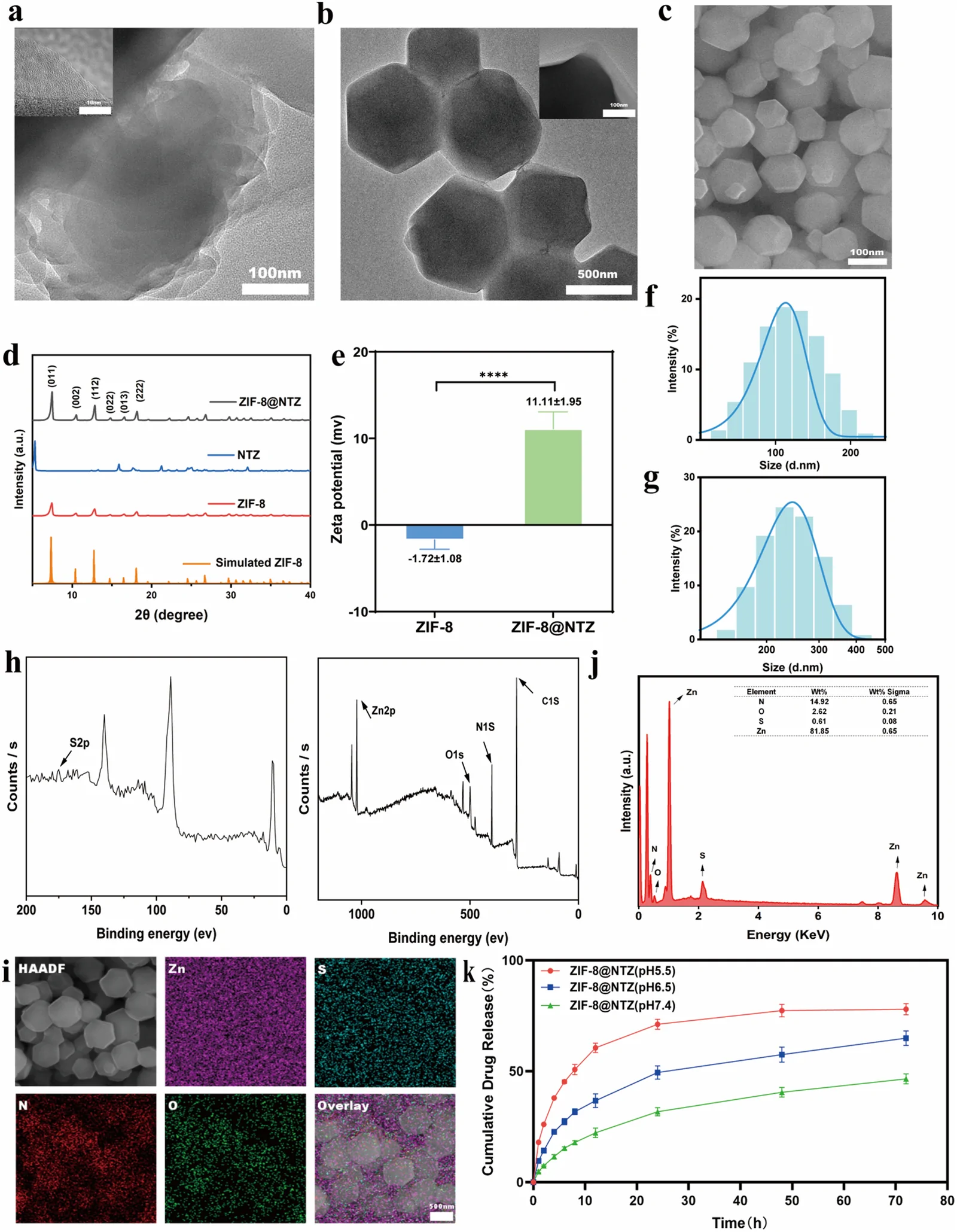
(a) High-resolution transmission electron microscopy (HRTEM) image of NTZ nanoparticles. (b) Transmission electron microscopy (TEM) image of ZIF-8@NTZ nanoparticles. (c) Scanning electron microscopy (SEM) image of ZIF-8@NTZ nanoparticles. (d) Powder X-ray diffraction (PXRD) patterns of ZIF-8, NTZ, and ZIF-8@NTZ nanoparticles. (e) Zeta potentials of ZIF-8 and ZIF-8@NTZ nanoparticles. (f) Particle size distribution of ZIF-8@NTZ. (g) Particle size distribution of ZIF-8. (h) X-ray photoelectron spectroscopy (XPS) of ZIF-8@NTZ nanoparticles. (i) Scanning transmission electron microscopy high-angle annular dark-field (STEM-HAADF) image of ZIF-8@NTZ nanoparticles and corresponding energy-dispersive spectroscopy (EDS) elemental mapping images of Zn, S, O, and N. (j) Elemental analysis of ZIF-8@NTZ nanoparticles. Energy-dispersive X-ray (EDX) spectrum of ZIF-8@NTZ nanoparticles. (k) Cumulative release of NTZ from ZIF-8@NTZ at different pH conditions.

We successfully synthesized and characterized ZIF-8@NTZ nanoparticles. High-resolution transmission electron microscopy (HRTEM) images showed that unencapsulated NTZ particles had clear internal lattice stripes, confirming that they were themselves well-crystallized nanocrystals. Transmission electron microscopy (TEM) images indicated that after ZIF-8 encapsulation, the particle size significantly increased, the perimeter became rounder, and the dispersion was uniform, successfully forming core (NTZ)-shell (ZIF-8) structured composite particles (Fig. 1b). Scanning electron microscopy (SEM) images further showed that ZIF-8@NTZ particles had regular rhombic dodecahedral morphology with a uniform coating layer on the surface, without obvious drug crystal exposure or aggregation (Fig. 1c). X-ray powder diffraction (PXRD) patterns provided key evidence at the crystal structure level. The pattern of ZIF-8@NTZ retained both the characteristic diffraction peaks of ZIF-8 and the main diffraction peaks of NTZ, with slightly reduced peak intensity, indicating that the encapsulation process did not disrupt the framework structure of ZIF-8 nor cause a polymorphic transformation of NTZ, successfully forming a stable composite system with coexisting crystal forms (Fig. 1d).

Nanoparticle size and zeta potential analysis showed that the surface potential of ZIF-8@NTZ changed from −1.72 ± 1.08 mV of ZIF-8 to +11.11 ± 1.95 mV (Fig. 1e), and this positive charge facilitates cellular uptake. Dynamic light scattering (DLS) results showed that the hydrated particle size of ZIF-8@NTZ was 231 nm (PDI < 0.2), significantly larger than that of ZIF-8 (109.5 nm), while maintaining a unimodal distribution, indicating that the drug was uniformly encapsulated without causing particle aggregation, providing an ideal size basis for its stable circulation *in vivo* and tumor enrichment (Fig. 1f and g). X-ray photoelectron spectroscopy (XPS) analysis showed characteristic peaks of C 1s, O 1s, N 1s, S 2p, and Zn 2p, consistent with the elemental composition of ZIF-8@NTZ (Fig. 1h). Energy-dispersive X-ray spectroscopy (EDS) element mapping clearly showed the distribution of Zn, S, O, and N elements, where the overlap area of Zn and N (from the ZIF-8 shell) and S (from the NTZ core) signals directly confirmed that ZIF-8 successfully grew on the NTZ surface, forming the expected core–shell structure (Fig. 1i and j). The final measured drug loading rate of ZIF-8@NTZ was as high as approximately 58.6%.

On this basis, the pH-responsive NTZ release behavior of ZIF-8@NTZ in PBS was studied at pH 5.5, 6.5, and 7.5 to determine its *in vitro* drug release rate. NTZ was rapidly released from ZIF-8@NTZ within the first 12 hours. The release ratio of ZIF-8@NTZ increased with decreasing pH, with NTZ almost completely released within 48 hours. After 48 hours, the cumulative release rate of NTZ at pH 5.5 was 78.7%, while at pH 6.5 and 7.4, the cumulative release rates were only 58.5% and 40.5%, respectively; this is beneficial for achieving targeted drug delivery at the tumor site (Fig. 1k).

### 3.2 *In vitro* antitumor activity of NTZ and ZIF-8@NTZ

**Fig. 2:**
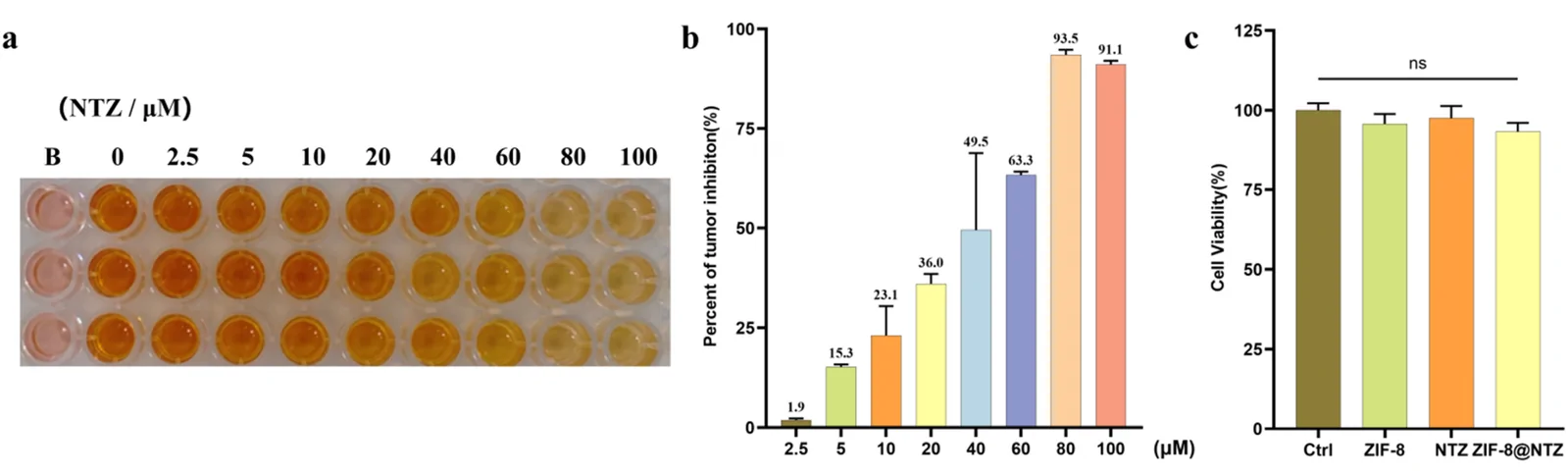
*In vitro* activity of NTZ in inhibiting cancer cells. (a) CCK8 assay was used to detect the HDST cell survival rate under NTZ treatment. (b) Statistical data showed that NTZ inhibited cancer cell activity at 40, 60, 80, and 100 µM concentrations (tumor growth inhibition rate exceeding 40% was considered effective). (c) Cell viability was assessed after 24 h treatment with Ctrl, ZIF-8, NTZ, or ZIF-8@NTZ. Data are presented as mean ± SD (*n* = 3). No significant difference (ns) was observed between groups (*p* > 0.05).

First, we evaluated the inhibitory capacity of NTZ on retinoblastoma cells. Through a high-throughput drug sensitivity test (HDST) using a patient-derived xenograft (PDX) model, the tumor tissues were treated with 2.5, 5, 10, 20, 40, 60, 80, and 100 µM respectively. The results showed that NTZ exhibited significant inhibitory effects on tumor tissues at approximately 40 µM concentration, and the inhibitory effect exhibited concentration dependence within a certain range (Fig. 2a and b).

**Fig. 3:**
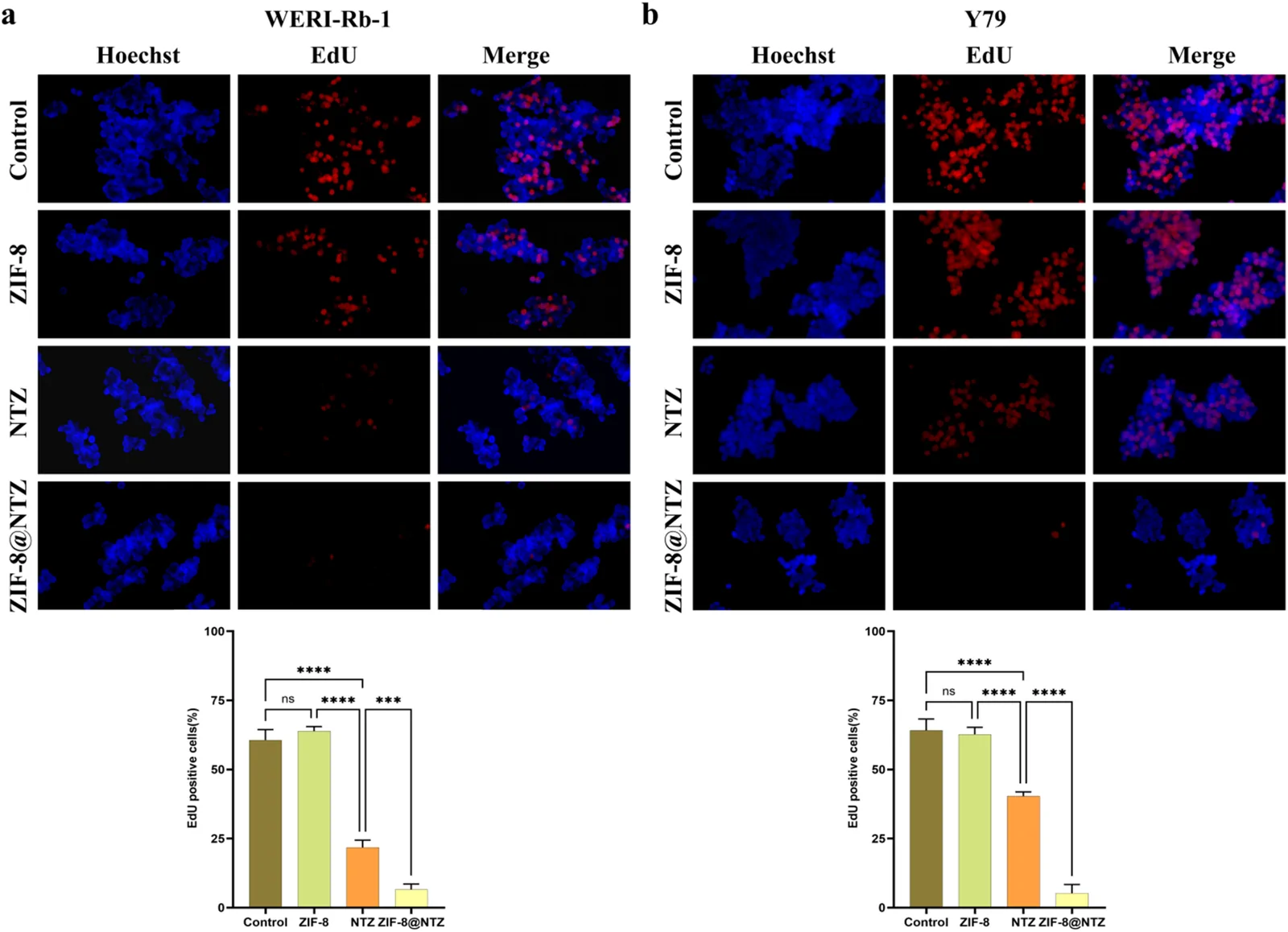
Cell EdU proliferation experiment. (a) WERI-Rb-1; (b) Y79. Hoechst staining total cell nuclei (blue), EdU labeling cells in S phase of proliferation (red), merge is the overlay image; control, NTZ, ZIF-8 and ZIF-8@NTZ groups sequentially showed that after NTZ was loaded onto ZIF-8, the WERI-Rb-1 cell EdU positive rate significantly decreased, scale bar = 20 µm.

The EdU cell proliferation experiment results showed that compared with the control group, free NTZ decreased the EdU-positive rate of Y79 cells; after being loaded with ZIF-8 (ZIF-8@NTZ), the inhibitory effect was further enhanced, significantly lower than that of free NTZ at the same dose (*P* < 0.01). A consistent trend was also observed in WERI-Rb-1 cells, where the EdU-positive rate in the ZIF-8@NTZ group was significantly lower than that in the free NTZ group and the control group. This fully demonstrates that the ZIF-8 nanocarrier can significantly enhance the inhibitory effect of NTZ on the proliferation of retinoblastoma cells (Fig. 3a and b).

**Fig. 4:**
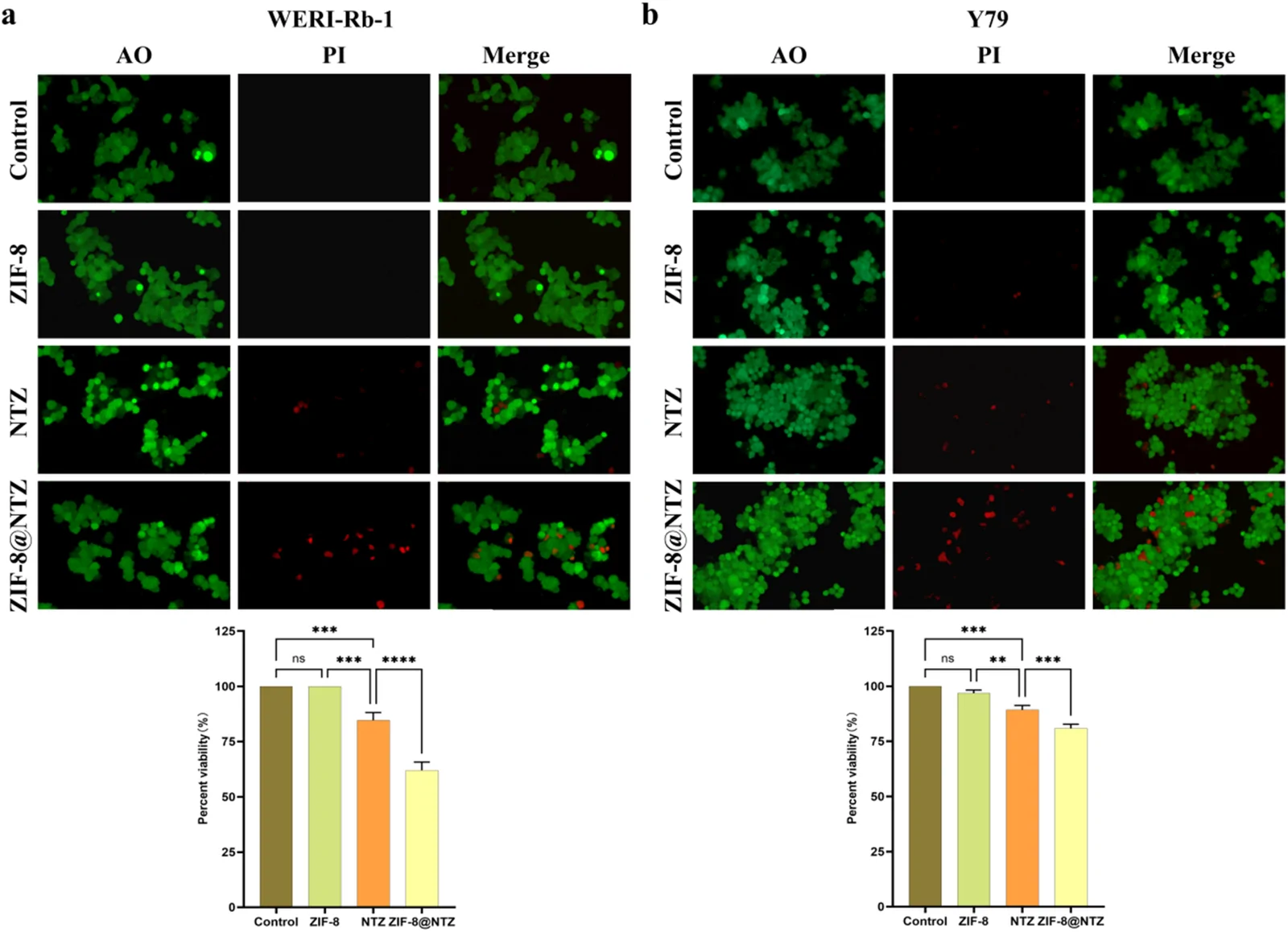
Live/dead cell staining in control, NTZ, ZIF-8 and ZIF-8@NTZ groups. (a) WERI-Rb-1; (b) Y79. Scale bar = 20 µm.

The live/dead cell staining experiment (AO/PI staining) showed that only a few/no cell death (red) was observed in the control group and blank ZIF-8 group, indicating that the ZIF-8 carrier itself has good biocompatibility. In contrast, a large number of dead cells appeared in the NTZ and ZIF-8@NTZ treatment groups, with the cell killing effect being most pronounced in the ZIF-8@NTZ group, where almost all cells were stained red by PI, further confirming its strong cytotoxicity (Fig. 4a and b).

**Fig. 5:**
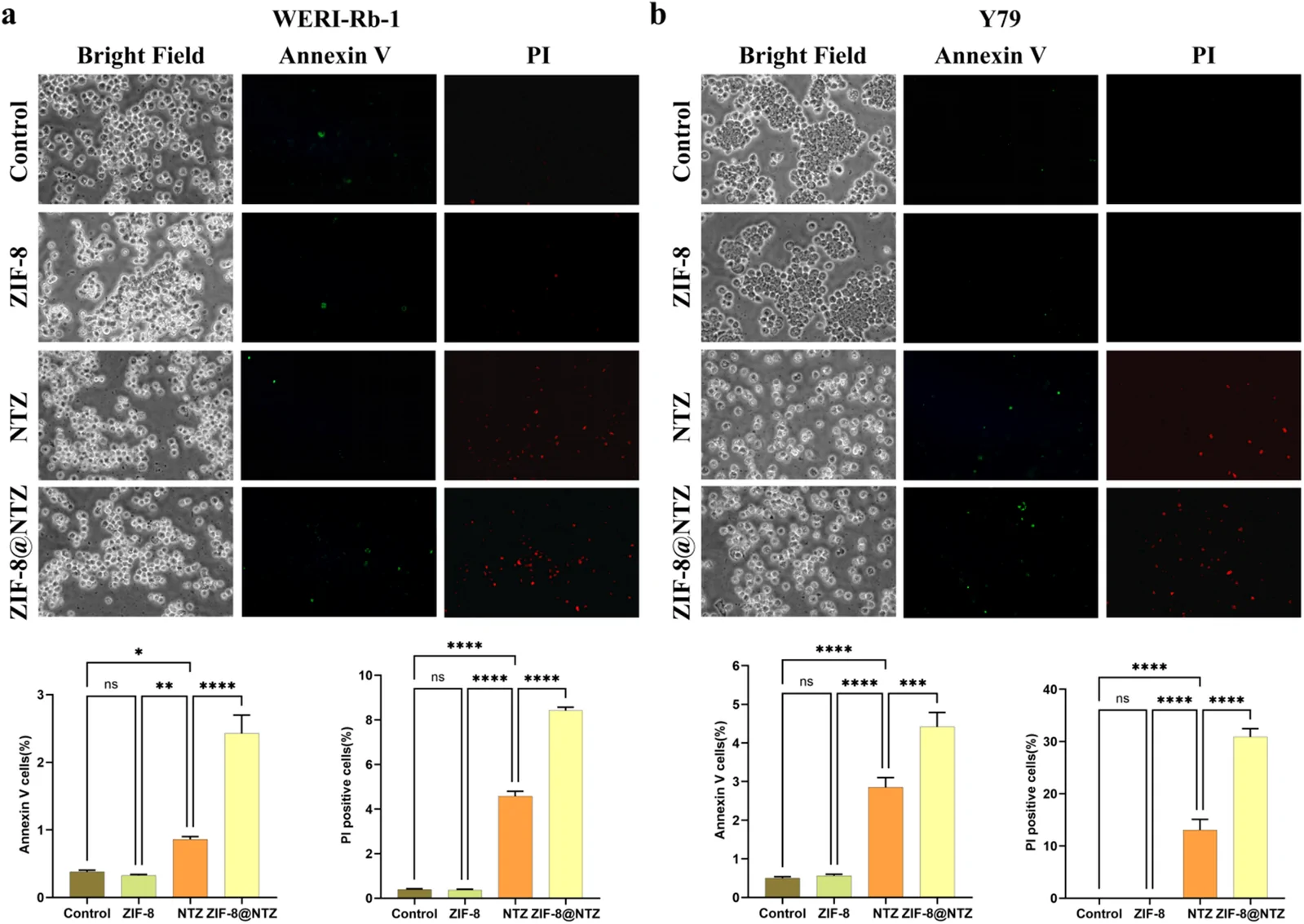
Apoptosis experiment in control, NTZ, ZIF-8 and ZIF-8@NTZ groups. (a) WERI-Rb-1, (b) Y79. Scale bar = 20 µm.

Further apoptosis detection (Annexin V-FITC/PI double staining) results corroborated the live/dead staining results. Except for a small number of apoptotic cells in the control group and ZIF-8 group, both NTZ and ZIF-8@NTZ groups induced a large number of cell apoptosis, and the pro-apoptotic effect was strongest in the ZIF-8@NTZ group, clarifying its mechanism of anti-tumor effect by inducing cell apoptosis (Fig. 5a and b).

### 3.3 Transcriptional analysis

**Fig. 6:**
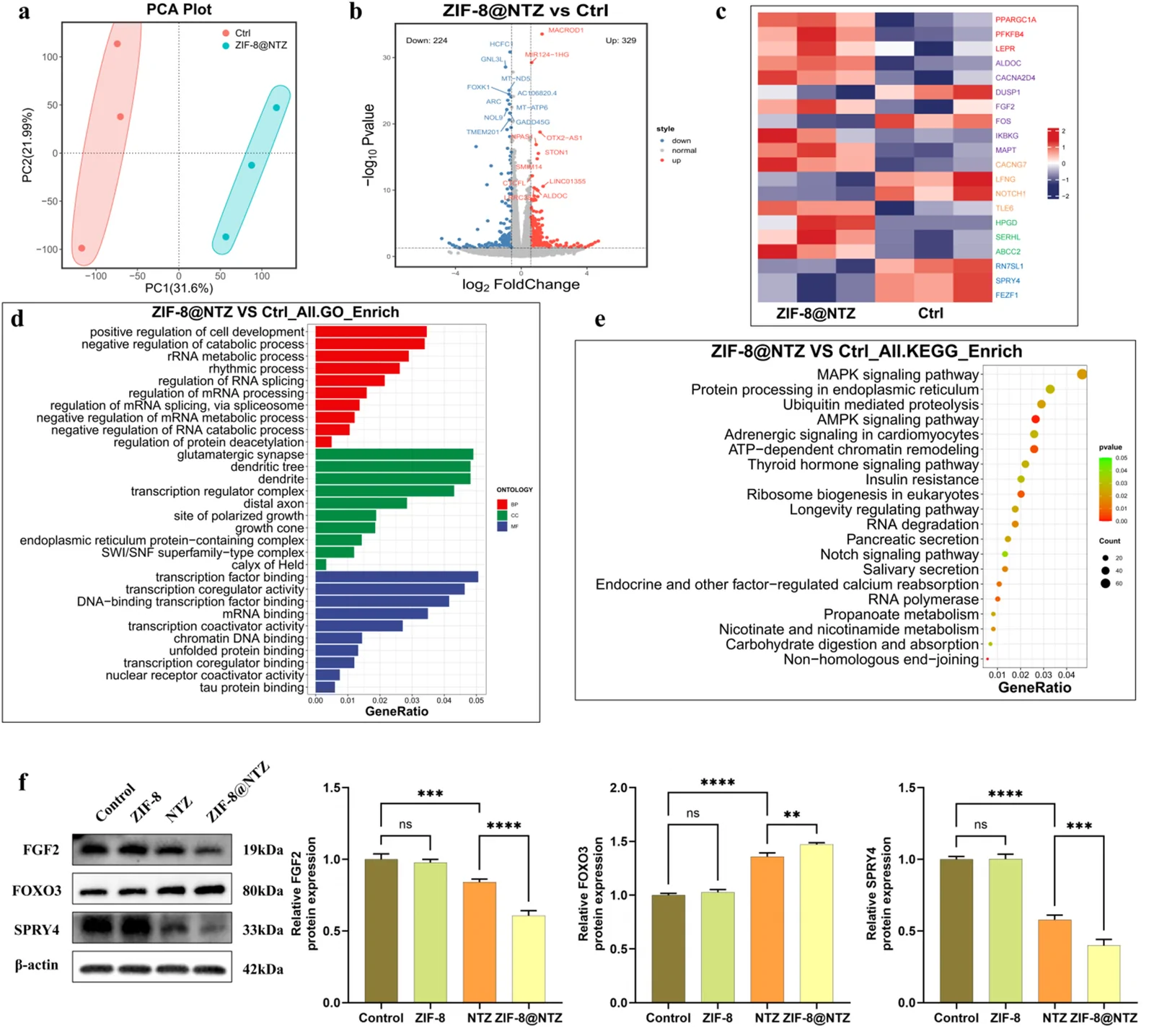
Transcriptome analysis after ZIF-8@NTZ treatment. (a) PCA analysis of ZIF-8@NTZ and control groups. (b) Volcano plot of DEGs between ZIF-8@NTZ group and control group. (c) Heatmap of DEGs related to AMPK pathway (red), MAPK pathway (purple), Notch pathway (red), immune infiltration and immune response (green), and invasion and migration (blue). (d) GO enrichment analysis of differentially expressed genes after different treatments. (e) KEGG pathway enrichment analysis of differentially expressed genes after different treatments. (f) Western blot analysis of protein levels (FGF2, FOXO3 and SPRY4) in WERI-Rb-1 cells.

To further investigate the effects of ZIF-8@NTZ treatment on gene expression patterns and potential molecular mechanisms, RNA-seq was performed on WERI-Rb-1 cells with or without ZIF-8@NTZ treatment. Principal component analysis (PCA) showed significant differences between the two groups after different treatments, facilitating further analysis (Fig. 6a). As shown in the volcano plot and differentially expressed genes (DEGs) heatmap (Fig. 6b), 329 genes were upregulated and 224 genes were downregulated in the ZIF-8@NTZ group. This indicates that ZIF-8@NTZ treatment significantly altered the mRNA expression profile of WERI-Rb-1 cells. Specifically, we observed significant differences in genes related to MAPK pathway, AMPK pathway, and Notch pathway. Meanwhile, genes specific to RB showed significant expression increases. Importantly, genes crucial for tumor malignancy, including genes related to cell proliferation, migration, and invasion, were significantly downregulated (RN7SL1, SPRY4, FEZF1) (Fig. 6c).

To elucidate the biological effects of DEGs, gene ontology (GO) enrichment analysis was performed. The enrichment analysis showed that ZIF-8@NTZ mainly affects cellular biological processes (*i.e.*, positive regulation of cell development and negative regulation of catabolic process), cellular components (*i.e.*, glutamatergic synapse), and molecular functions (*i.e.*, transcription factor binding) (Fig. 6d). Additionally, the Kyoto Encyclopedia of Genes and Genomes (KEGG) pathway database was used to study the specific signaling pathways involved in DEGs. ZIF-8@NTZ upregulated MAPK signaling pathway and AMPK signaling pathway to mediate cell apoptosis, while reducing tumor cell migration and invasion and alleviating the immunosuppressive environment (Fig. 6e). The transcriptome analysis showed that ZIF-8@NTZ significantly inhibited tumor cell invasion and migration. To further explore the potential molecular mechanism underlying the anti-tumor effect of ZIF-8@NTZ, western blotting (WB) assay was performed to verify the expression levels of core proteins associated with the MAPK signaling pathway, as well as key molecules governing tumor cell migration and invasion. Consistent with the transcriptomic profiling results, WB validation demonstrated that ZIF-8@NTZ markedly regulated the activation of the MAPK signaling cascade and modulated the expression of migration-related proteins (Fig. 6f).

### 3.4 *In vivo* anti-tumor efficacy of ZIF-8@NTZ

**Fig. 7:**
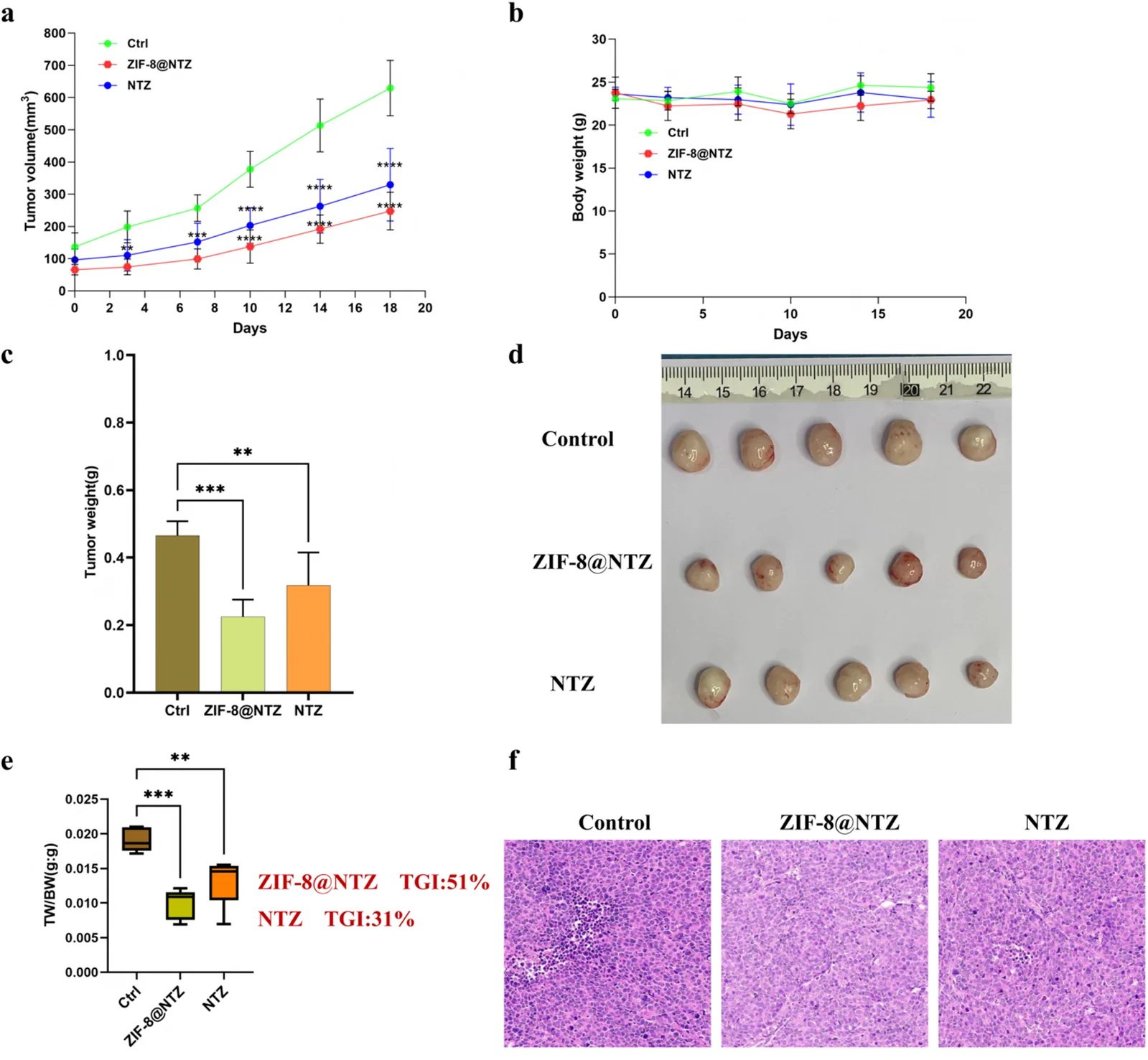
ZIF-8@NTZ effectively inhibits cancer *in vivo*. (a and b) Changes in tumor volume and mouse body weight after ZIF-8@NTZ inhibition. (c) Statistical chart of ZIF-8@NTZ inhibiting tumor growth. (d) Tumor photos after mice were euthanized. (e) Mouse tumor weight/body weight ratio (TW/BW) chart, with TGI calculated. (f) Representative H&E staining images of tumor tissues.

To evaluate the *in vivo* efficacy of ZIF-8@NTZ, we constructed a subcutaneous xenograft model of retinoblastoma. Twenty-four tumor-bearing nude mice were randomly divided into three groups: blank control group, ZIF-8 group, NTZ group, and ZIF-8@NTZ group. After 28 days of drug treatment, the results showed that compared with the control group, both NTZ and ZIF-8@NTZ groups could effectively inhibit tumor growth, among which the anti-tumor effect of the ZIF-8@NTZ group was the most significant. Both tumor volume and final tumor weight were significantly smaller than the other two groups, while there was no significant difference in body weight (Fig. 7a–e). H&E staining showed that the control group exhibited densely packed viable tumor cells with deeply stained nuclei and a high nuclear-to-cytoplasmic ratio. NTZ treatment induced moderate histological changes, including reduced cell density, increased cellular heterogeneity, and scattered nuclear pyknosis and karyorrhexis, while many viable tumor cells remained. In contrast, ZIF-8@NTZ caused marked tumor tissue destruction, characterized by significantly decreased cell density, disrupted tissue architecture, enlarged intercellular spaces, and frequent nuclear pyknosis and karyorrhexis, indicating enhanced antitumor efficacy (Fig. 7f).

### 3.5 *In vivo* biosafety assessment of ZIF-8@NTZ

H&E staining of the major organs (heart, liver, kidney, and spleen) revealed no obvious histopathological abnormalities in the control, NTZ, or ZIF-8@NTZ groups. No evidence of tissue injury, inflammatory infiltration, necrosis, or structural damage was observed, indicating that ZIF-8@NTZ exhibited favorable *in vivo* biosafety.

## 4. Discussion

In this study, we successfully constructed a ZIF-8-based nanodelivery system (ZIF-8@NTZ) for efficient loading of the hydrophobic anti-tumor drug NTZ, and systematically evaluated its application potential in the treatment of RB. The research results consistently showed that ZIF-8@NTZ significantly enhanced the *in vitro* and *in vivo* anti-tumor efficacy of NTZ through its unique physicochemical properties and intelligent drug release behavior.

First, we fully characterized the successful construction of ZIF-8@NTZ nanoparticles. HRTEM, TEM, and SEM images clearly confirmed the formation of a core–shell structure, where crystalline NTZ was completely encapsulated by the ZIF-8 framework. The coexistence of characteristic peaks of ZIF-8 and NTZ in the PXRD pattern confirmed that the coating process did not destroy the drug crystal form, while maintaining the integrity of the ZIF-8 skeleton, which is consistent with the strategy of one-step synthesis of high drug loading ZIF-8 reported by Li *et al.*^27^ The final particle size (∼231 nm) and positive surface charge (+11.11 mV) of the nanoparticles are very conducive to enrichment in tumor tissues through the EPR effect and promoting interaction with negatively charged cell membranes.^28^ Because frequent intravitreal injections may induce endophthalmitis and vitreous hemorrhage, a drug should use a delivery system with lower complication risk and higher efficacy for chemotherapy.^29^

Most importantly, ZIF-8@NTZ exhibited significant pH-responsive drug release properties, with the cumulative drug release (78.7%) under simulated tumor microenvironment (pH 5.5) being much higher than that in physiological environment (pH 7.4, 40.5%). This “smart” drug release behavior is mainly attributed to the instability of the ZIF-8 framework under acidic conditions, and its mechanism is highly consistent with the research results of Li *et al.*^30^ This characteristic ensures stable drug circulation in the blood and efficient release at the tumor site, which is key to achieving targeted therapy and reducing systemic toxicity.

At the cellular level, this study confirmed that NTZ itself has a concentration-dependent inhibitory effect on RB cells, which echoes the mechanism found by Sun *et al.* in bladder cancer where NTZ exerts anti-tumor effects by activating the ROS-p53 pathway.^31^ However, the core finding of this study is that the ZIF-8 nanocarrier greatly enhances the efficacy of NTZ. The EdU experiment showed that the inhibitory capacity of ZIF-8@NTZ on cell proliferation was significantly stronger than that of free NTZ. We speculate that its enhancement mechanism may stem from two aspects: first, ZIF-8 nanoparticles promote the endocytosis of drugs by cells, increasing intracellular drug concentration; second, the Zn^2+^ produced by ZIF-8 degradation can induce elevated intracellular reactive oxygen species (ROS) levels, which may have a synergistic effect with the inherent ability of NTZ to activate the ROS-p53 pathway, thereby amplifying its pro-apoptotic signals.^32^ This hypothesis of synergistic enhancement was further verified in subsequent live/dead staining and cell apoptosis experiments. ZIF-8@NTZ showed the strongest cell killing and pro-apoptotic effects, while the blank ZIF-8 group showed good biocompatibility, indicating that the carrier itself is safe and its enhancement effect mainly comes from drug delivery efficiency and possible synergistic effects. These results strongly indicate that loading NTZ into ZIF-8 not only solves the delivery bottleneck of its poor water solubility but may also activate stronger anti-tumor pathways through metal ion synergy. *In vivo* anti-tumor experiments provide the most convincing evidence for the translational potential of ZIF-8@NTZ.^33^

In the PDX model of RB, the tumor growth inhibition effect of the ZIF-8@NTZ group was significantly superior to that of the free NTZ group. This is undoubtedly the embodiment of its excellent *in vitro* performance *in vivo*: the nanometer size enables passive targeting of tumor tissue through the EPR effect, the acidic microenvironment triggers drug “burst release,” and the possible synergistic effect of Zn^2+^ jointly lead to high drug concentration and potent killing at the tumor site.^34^ This finding is significant as it provides a new approach to overcoming the blood-retinal barrier (BRB), the main obstacle in treating RB. Although this study used a subcutaneous xenograft model, the ZIF-8 nanosystem also has application prospects in intraocular administration (such as intravitreal injection), and its controllable size and sustained-release characteristics are expected to reduce the risks associated with frequent injections. As emphasized by Lee *et al.*, nanotechnology is an effective strategy for overcoming intraocular drug delivery barriers.^35^ Of course, this study also has some limitations. First, the specific molecular mechanism of the synergistic effect between Zn^2+^ and NTZ has not been thoroughly explored. Second, the current research is mainly based on subcutaneous tumor models. Third, future studies need to further verify the targeting and efficacy of ZIF-8@NTZ in intraocular orthotopic RB models and systematically evaluate its systemic and intraocular safety.

## 5. Conclusions

This study successfully designed and synthesized a ZIF-8 core–shell nanocarrier (ZIF-8@NTZ) for loading nitazoxanide (NTZ). The carrier has high drug loading capacity, good stability, and significant pH-responsive drug release characteristics. *In vitro* experiments have shown that ZIF-8@NTZ can effectively inhibit the proliferation of retinoblastoma cells and induce their apoptosis, with effects significantly superior to free NTZ. *In vivo* animal experiments further confirmed that ZIF-8@NTZ can efficiently target tumor tissue and significantly inhibit tumor growth. In summary, the ZIF-8@NTZ nano-delivery system provides an effective strategy to solve the problems of poor water solubility and low bioavailability of NTZ, offering a new potential option for the targeted treatment of retinoblastoma.

## Ethics approval

All animal experiments were performed in accordance with the guidelines of the Animal Protection Committee of Nanchang University and approved by the Institutional Animal Care and Use Committees of the First Affiliated Hospital of Nanchang University.

## Author contributions

M. Q.: conceptualization, investigation, methodology, and writing – original draft. S. X.: conceptualization, investigation, methodology, and writing – review and editing. R. Z.: investigation and visualization. Y. S.: language, conceptualization, resources, and project administration. J. Z.: conceptualization, supervision, review and editing, and funding acquisition. All authors read and approved the final manuscript.

## Conflicts of interest

The authors declare that they have no known competing financial interests or personal relationships that could have appeared to influence the work reported in this paper.

## Data Availability

All data generated or analyzed during this study are included in this published article and will be available from the corresponding author upon reasonable request. Supplementary information (SI) is available. See DOI: https://doi.org/10.1039/d6ra04041a.
